# Paternal age and neonatal outcomes: a population-based cohort study

**DOI:** 10.1093/hropen/hoaf006

**Published:** 2025-02-26

**Authors:** Wenxue Xiong, Xijia Tang, Lu Han, Li Ling

**Affiliations:** Department of Medical Statistics, School of Public Health, Sun Yat-Sen University, Guangzhou, Guangdong, China; Department of Medical Statistics, School of Public Health, Sun Yat-Sen University, Guangzhou, Guangdong, China; National Health Commission Key Laboratory of Male Reproduction and Genetics, Guangdong Provincial Reproductive Science Institute (Guangdong Provincial Fertility Hospital), Guangzhou, China; Department of Medical Statistics, School of Public Health, Sun Yat-Sen University, Guangzhou, Guangdong, China; Clinical Research Design Division, Clinical Research Center, Sun Yat-Sen Memorial Hospital, Sun Yat-Sen University, Guangzhou, Guangdong, China

**Keywords:** advanced paternal age, caesarean section, preterm birth, neonatal outcomes, preconception care

## Abstract

**STUDY QUESTION:**

Is paternal age associated with neonatal outcomes?

**SUMMARY ANSWER:**

Paternal age is independently associated with preterm birth (PTB) and caesarean section.

**WHAT IS KNOWN ALREADY:**

Advanced maternal age has long been recognized as a major risk factor for adverse neonatal outcomes. However, the association between paternal age and neonatal outcomes are not well established, yet it is biologically plausible that an increasing number of genetic and epigenetic sperm abnormalities in older males may contribute to adverse neonatal outcomes.

**STUDY DESIGN, SIZE, DURATION:**

This population-based cohort study was based on the National Free Preconception Checkups Project between 1 January 2014 and 31 December 2019 in Guangdong Province, China. Paternal age at the maternal last menstrual period was measured. The main outcomes included caesarean section, PTB, small for gestational age (SGA) and perinatal infant death (PID).

**PARTICIPANTS/MATERIALS, SETTING, METHODS:**

A total of 783 988 mother–neonate–father trios were included in this study. A modified Poisson regression model was employed to estimate relative risk (RR) and 95% CI and logistic regression models were used to analyse the relative importance of predictors. We used restricted cubic splines to flexibly model the non-linear dose–response association between paternal age and neonatal outcomes. We also assessed additive interactions between paternal and maternal age on neonatal outcomes.

**MAIN RESULTS AND THE ROLE OF CHANCE:**

Neonates born to fathers aged 35–44 years had higher risks of caesarean section (RR: 1.07; 95% CI: 1.06–1.09) and PTB (RR: 1.15; 95% CI: 1.10–1.19) compared with neonates of fathers aged 25–34 years, after adjustment for confounders. The increased risks of PTB associated with paternal age appeared to be ‘dose’ dependent, with a J-shaped association curve (*P* for non-linearity<0.001). The relative importance of paternal age in predicting PTB and caesarean section was similar to, or even higher than, that of maternal age. The combined effects of advanced maternal and paternal age appeared to be less than additive joint effects (relative excess risk due to interaction<0). The association of paternal age with SGA or PID was not statistically significant (*P *>* *0.05).

**LIMITATIONS, REASONS FOR CAUTION:**

As with all observational studies, residual confounding could not be ruled out. Only couples who planned to conceive were included.

**WIDER IMPLICATIONS OF THE FINDINGS:**

In this population-based cohort study, paternal age was independently associated with caesarean section and PTB. These findings may be clinically useful in preconception counselling on parental age-related pregnancy risks. Our findings emphasize the need to further investigate the public health implications of increasing paternal age.

**STUDY FUNDING/COMPETING INTEREST(S):**

This study was supported by the Guangdong Province Medical Research Funding (No. B2023416). No competing interests are reported.

**TRIAL REGISTRATION NUMBER:**

N/A.

WHAT DOES THIS MEAN FOR PATIENTS? The age at which couples have children has been gradually delayed worldwide, and advanced maternal age has long been recognized as a major risk factor for adverse neonatal outcomes. However, the association between paternal age and neonatal outcomes are not well established, yet it is biologically plausible that an increasing number of genetic and epigenetic sperm abnormalities in older males may contribute to adverse neonatal outcomes.

Using data of 783 988 mother–neonate–father trios from the National Free Preconception Health Examination Project (2014–2019) in Guangdong Province, China, this large population-based cohort study investigated the association between paternal age and neonatal outcomes. We found that paternal age is associated with preterm birth and caesarean delivery, even after taking into account possible confounding factors, and the relative importance of paternal age in predicting preterm birth and caesarean delivery was similar to, or even higher than, that of maternal age. The association of paternal age with small for gestational age, or perinatal infant death was not statistically significant in this study. This study underscores the importance of including, in reproductive life plans, discussions of paternal age and other risk factors that are related to age.

## Introduction

Globally, the phenomenon of couples having children later in life has increased during the past several decades (Khandwala *et al.*, 2017; du Fossé *et al.*, 2020). Advanced maternal age has long been recognized as a major risk factor for adverse neonatal outcomes (Cleary-Goldman *et al.*, 2005; Wang *et al.*, 2020). However, the association between paternal age and neonatal outcomes is not well established, yet it is biologically plausible that an increasing number of genetic and epigenetic sperm abnormalities in older males may contribute to adverse neonatal outcomes (Sartorius and Nieschlag, 2010; Nybo Andersen and Urhoj, 2017). Indeed, the emerging Paternal Origins of Health and Disease paradigm posits that paternal factors, such as paternal age, can modify the sperm epigenome, yielding epigenetic changes that are maintained in the offspring, in whom they may affect gene regulation and physiology (Soubry, 2018). A better understanding of the preconception origins of disease through the paternal exposome will ultimately help instruct and guide public health policies in the future (Soubry, 2018).

However, previous research on the impact of paternal age on the health of offspring has been limited mostly to the risk of congenital diseases and has been conducted in developed countries (Su *et al.*, 2015; Nybo Andersen and Urhoj, 2017; Brandt *et al.*, 2019). Some studies have investigated whether paternal age is associated with neonatal outcomes, such as preterm birth (PTB) and small for gestational age (SGA), but their results were inconsistent (Zhu *et al.*, 2005; Alio *et al.*, 2012; Hurley and DeFranco, 2017). These studies all treated paternal age as a categorical variable and used different cut-offs, which could lead to information loss, power reduction and complexities in analysis (Altman and Royston, 2006). Instead of categorizing continuous variables, restricted cubic spline (RCS) functions can characterize a non-linear dose–response relationship between a continuous exposure and an outcome (Desquilbet and Mariotti, 2010). The joint effect of paternal and maternal age on neonatal outcomes also has not been well investigated (Yin *et al.*, 2024). In addition, inference from previous studies is also challenging because they have used logistic regression models to estimate the odds ratio (OR) to approximate the relative risk (RR). However, when outcomes are common, the estimated OR is not close to the RR and will overestimate the association (McNutt *et al.*, 2003; Richardson *et al.*, 2015). In the context of changes in the birth policy in China, more and more couples are having children at an older age (Li *et al.*, 2019). However, only a few hospital-based studies have explored the association of paternal age with neonatal outcomes among the Chinese population (Mao *et al.*, 2021; Mao *et al.*, 2022; Yin *et al.*, 2024). The homogeneity of the hospital-based cohort may decrease the external validity of the conclusions (Mao *et al.*, 2022).

Therefore, we conducted this population-based cohort study among over 0.7 million mother–neonate–father trios based on the National Free Preconception Checkups Project (NFPCP) in Guangdong Province, China, to elucidate the association between paternal age and neonatal outcomes. We also investigated the joint association of paternal and maternal age with neonatal outcomes.

## Materials and methods

### Study design

This population-based, retrospective cohort study was conducted by the NFPCP in Guangdong Province, China, a free health service for childbearing-aged couples who plan to conceive. Couples who had made their conception plans were volunteering to participate in the NFPCP. The detailed design, organization and quality control have been published previously (Zhou *et al.*, 2016; Xiong *et al.*, 2022; Xiong *et al.*, 2024). Briefly, there were 3 main phases in which detailed information was collected in the NFPCP: preconception health examinations, early pregnancy follow-up, and pregnancy outcome follow-up. During the preconception health examinations stage, trained local health workers used a standardized questionnaire through a face-to-face interview to collect the couples’ respective baseline information, including demographic characteristics (birth date, educational level, and economic insecurity), history of chronic diseases, lifestyle (alcohol intake and tobacco use), and women’s history of pregnancy. Then, experienced health workers conducted medical examinations, including measuring bodyweight and height. BMI was calculated as the body weight (kilograms) divided by the square of the height (meters). Blood samples (5 ml) were collected after a fast of at least 8 h. Samples were stored at 4–8°C and transported for analysis within 24 h. Serum fasting plasma glucose (FPG) concentration was measured using the glucose oxidase method in the local laboratories.

During the early pregnancy follow-up stage, participants were followed up by trained health workers by telephone or face-to-face within 3 months after the preconception health examination was completed to update pregnancy status and collect the date of the last menstrual period (LMP). During the pregnancy outcome follow-up stage, participants who became pregnant were contacted within 1 year after the early pregnancy follow-up to ascertain pregnancy outcomes by the local trained interviewers. Information regarding current pregnancy outcomes (babies with no obvious abnormalities detected on examination at birth, PTB, low birth weight, birth defect, miscarriage, miscarriage, induced abortion, ectopic pregnancy or stillbirth), neonate’s sex, birth weight (in grams), neonatal information (singleton or multiple births), delivery date, gestational weeks, place of delivery, delivery mode (caesarean delivery, assisted vaginal delivery or spontaneous vaginal birth) and infant survival at 42 days was collected from the medical records at local hospitals. A web-based health services information system included all the information obtained above. The National Center of Clinical Laboratories for Quality Inspection and Detection was responsible for the external quality assessment and for quality control.

### Study population

From 1 January 2014 to 31 September 2019, a total of 881 369 couples had singleton birth records in the NFPCP in Guangdong Province. To control for the effect of multiple births, we did not include 7391 multiple pregnancies, as in previous studies (Liu *et al.*, 2017; Wang *et al.*, 2018). We then excluded 89 024 couples with missing information at baseline and early pregnancy follow-up, 4868 couples with missing information on paternal age, and 3489 couples with other adverse pregnancy outcomes (including ectopic pregnancy and therapeutic or medically induced abortion). The final analysis included 783 988 mother–neonate–father trios (Supplementary Fig. S1). The NFPCP was approved by the Institutional Review Board of the Chinese Association of Maternal and Child Health Studies and all participants provided a written informed consent form before enrolment. This study followed the Strengthening the Reporting of Observational Studies in Epidemiology reporting guideline.

### Ascertainment of outcomes

A literature review was conducted to determine neonatal outcomes that have previously been associated with paternal age (Sartorius and Nieschlag, 2010; Alio *et al.*, 2012; Hurley and DeFranco, 2017; Nybo Andersen and Urhoj, 2017; Khandwala *et al.*, 2018; Brandt *et al.*, 2019). Of these variables, those available within the NFPCP data files were included: (i) caesarean delivery, (ii) PTB (babies born alive before 37 weeks of pregnancy are completed), (iii) SGA (birth weight by gestational age and sex below the 10th percentile according to Chinese birthweights) (Zhu *et al.*, 2015), and (iv) perinatal infant death (PID, stillbirth after 28 weeks of gestation or newborns who died after birth within 7 days). All outcomes were categorized as dichotomous, with gestational age (weeks) and birth weight (grams) also presented as continuous variables.

### Covariates definition

A literature review was conducted and we used the directed acyclic graph approach to select the confounders for adjustment in multivariable models. We constructed the graph using online DAGitty version 3.1 to identify minimally sufficient adjustment sets of covariates (Textor *et al.*, 2016). We included the following variables in multivariable models: couple’s economic pressure (none, a little, or a lot), maternal age at LMP (continuous), maternal higher education (defined as senior high school, college, or higher, yes/no) and first gestation (yes/no) (Supplementary Fig. S2). In the sensitivity, we also included maternal BMI (continuous), alcohol intake (no drinking, occasional, or frequent), tobacco exposure (tobacco use or exposure to passive smoking, yes/no), diabetes (self-reported history of diabetes or FPG ≥7.0 mmol/l, yes/no), paternal BMI (continuous), alcohol intake (no drinking, occasional, or frequent) and tobacco use (no smoking, 1–9, 10–19, or ≥20 per day), to test the robustness of the results. We used the variance inflation factor to measure the potential multicollinearity problem for covariates, and all variance inflation factors were <3 in this study.

### Statistical analyses

Mean (SD) and number (percentages) were used to describe the baseline characteristics of participants. Consistent with a previous study (Khandwala *et al.*, 2018), paternal age at maternal LMP was first categorized into 4 intervals: ≤24, 25–34, 35–44, and ≥45 years, with fathers aged 25–34 years as the reference group. To quantify the effect of paternal age on the dichotomous outcomes of adverse neonatal outcomes, we used modified Poisson regression with robust (sandwich) estimation of variance to estimate unadjusted and adjusted RR and 95% CI (Zou, 2004). Linear models were employed to evaluate the association of paternal age with gestational age and birth weight. The non-linear dose–response relationship of paternal age and risk of adverse neonatal outcomes were assessed using RCS based on logistic regression models. The non-linearity trend was tested by the Wald statistics.

In *post hoc* analyses, we estimated how important paternal age is in predicting caesarean delivery and PTB by calculating the standardized coefficients of paternal age and other covariates based on the fully adjusted logistic model (Menard, 2004; Wu *et al.*, 2021). Taking the absolute value of the standardized coefficients enables them to be ranked from highest to lowest in order of strength of association with the outcome (Thompson, 2009). We tested the consistency of the results by modelling a separate logistic model for each predictor to estimate the c‐statistic for each model, then ranking the predictors in terms of the absolute value of their c‐statistic difference from 0.5 (Thompson, 2009).

To explore the impact of subfertility, we assessed the associations across time to pregnancy (TTP) in the subgroup analysis. The TTP was calculated (date of LMP−date of baseline questionnaire completion), and couples were grouped into 2 groups: <12 or ≥12 months (DoPierala *et al.*, 2016). We also investigated the associations of paternal age with caesarean delivery and PTB, stratified by maternal age at LMP (<25, 25–34, and ≥35 years) (Khandwala *et al.*, 2018). In addition, we evaluated whether the combined effects of paternal and maternal age on caesarean delivery and PTB were more or less additive, which was considered to be most appropriate for assessing the public health importance of interactions (Knol and VanderWeele, 2012). The possible additive interaction was measured by relative excess risk due to interaction (RERI) (Andersson *et al.*, 2005; Sjölander, 2018). When RERI is equal to 0, it indicates simple additive risks (i.e. the absence of an additive interaction); an RERI greater than 0 denotes a synergetic interaction; and an RERI <0 indicates less than additive joint effects. In order to present the magnitude of the joint associations in a more straightforward manner, we categorized maternal and paternal age into 2 binary variables (mother: <35 or ≥35 years; father: <35 or ≥35 years). Most studies denote paternal age of 40 years or more as fathers of advanced age, but the risk increase in indisputable paternal age-related conditions starts around the paternal age of 35 years (Crow, 2000; Nybo Andersen and Urhoj, 2017). We chose the stratum with the lowest risk as the reference category, i.e. both mother and father aged <35 years (Knol *et al.*, 2011). The calculation of RERI was as follows: RERI = RR_mother≥35 and father≥35_ − RR_mother≥35 and father<35_−RR_mother<35 and father≥35_ + 1 (Knol *et al.*, 2011).

In the primary analysis, we assumed that missing covariates were missing completely at random, then the complete case analysis was performed (Supplementary Table S1). In the sensitivity analysis, we used multiple imputation by chained equations to impute missing covariates with 10 data sets, and combined the results on each complete data set using Rubin’s rule (Rubin and Schenker, 1991). All variables in the multivariable model, including outcomes, were included in the multiple imputation model (Sterne *et al.*, 2009). We conducted negative control outcome analysis to further evaluate the potential bias due to unmeasured confounding. Negative control outcomes are required to be causally unrelated to the exposure variable (Lousdal *et al.*, 2020). Therefore, we considered secondary sex ratio as the negative control outcome, which is defined as the ratio of the number of boys to girls at birth and was unlikely to be associated with paternal age according to any known pathophysiologic mechanisms (Sartorius and Nieschlag, 2010; Nybo Andersen and Urhoj, 2017; Khandwala *et al.*, 2018). Thus, an association between paternal age and secondary sex ratio may suggest the presence of uncontrolled confounders. We also calculated the E-value to explore the minimum strength of the association that an unmeasured confounder would need to have with both paternal age and neonatal outcomes to fully explain away the estimated association, conditional on the measured covariates (VanderWeele and Ding, 2017).

Analyses were performed using SAS version 9.4 (SAS Institute Inc., Cary, NC, USA) and R software version 4.3.2 (R Foundation for Statistical Computing, Vienna, Austria) with the analysis packages tableone, version 0.13.2; sandwich, version 3.0–2; rms, version 6.3–0; and mice, version 3.15.0. We considered the 2-sided test of *P*<0.05 to be statistically significant.

## Results

A total of 783 988 men (mean [SD] age, 28.9 [4.8] years) and their female partners (26.7 [4.3] years) with singleton births were included in the final analysis. Paternal and maternal demographic characteristics by paternal age group are shown in Table 1. The majority (71.1%) of fathers were 25–34 years old when their female partners became pregnant, and as paternal age increased, the corresponding maternal age also increased.

**Table 1.** Maternal, paternal, and neonatal characteristics by paternal age group.

	Overall	≤24	25–34	35–44	≥45
	(n = 783 988)	(n = 130 219)	(n = 557 671)	(n = 90 670)	(n = 5428)
**Maternal characteristic**					
Age at LMP (SD), years	26.7 (4.3)	22.8 (1.9)	26.4 (3.2)	33.5 (4.1)	36.7 (5.2)
Higher education, No. (%)	430 507 (62.2)	58 815 (50.1)	314 118 (63.7)	54 659 (71.2)	2915 (64.7)
BMI (SD), kg/m^2^	20.8 (2.9)	20.4 (2.7)	20.7 (2.9)	21.9 (3.1)	22.4 (3.2)
Diabetes mellitus, No. (%)	11 912 (1.6)	1783 (1.4)	8340 (1.6)	1639 (1.9)	150 (2.9)
Alcohol intake, No. (%)	50 084 (6.5)	5647 (4.4)	36 863 (6.8)	7153 (8.1)	421 (8.0)
Tobacco exposure, No. (%)	140 521 (18.3)	18 063 (14.1)	102 464 (18.8)	18 945 (21.4)	1049 (19.9)
First gestation, No. (%)	495 552 (63.2)	101 004 (77.6)	371 396 (66.6)	21 708 (23.9)	1444 (26.6)
**Paternal characteristic**					
BMI (SD), kg/m^2^	22.6 (3.2)	21.8 (3.1)	22.6 (3.2)	23.6 (3.2)	23.7 (2.9)
Alcohol intake, No. (%)	275 039 (36.2)	43 320 (34.0)	196 053 (36.3)	33 826 (39.0)	1840 (36.0)
Tobacco use, No. (%)	216 793 (28.5)	41 829 (32.8)	148 309 (27.4)	25 156 (29.0)	1499 (29.3)
Couple’s economic pressure, No. (%)					
No	409 052 (53.8)	75 637 (59.5)	288 216 (53.3)	42 369 (48.7)	2830 (55.2)
A little	319 873 (42.1)	48 361 (38.0)	228 682 (42.3)	40 698 (46.8)	2132 (41.6)
A lot	31 041 (4.1)	3205 (2.5)	23 705 (4.4)	3964 (4.6)	167 (3.3)
**Neonatal outcomes**					
Boy birth, No. (%)	410 996 (52.4)	68 122 (52.3)	291 872 (52.3)	48 084 (53.0)	2918 (53.8)
Caesarean delivery, No. (%)	173 459 (22.1)	18 296 (14.1)	116 931 (21.0)	35 871 (39.6)	2361 (43.5)
Preterm birth, No. (%)	40 859 (5.2)	6981 (5.4)	28 152 (5.0)	5350 (5.9)	376 (6.9)
Gestational age (SD), weeks	38.94 (1.77)	39.02 (1.82)	38.97 (1.76)	38.70 (1.73)	38.59 (1.86)
Small for gestational age, No. (%)	66 515 (8.5)	11 395 (8.8)	47 974 (8.6)	6748 (7.4)	398 (7.3)
Birth weight (SD), grams	3183.4 (384.1)	3170.9 (359.5)	3183.2 (384.0)	3201.1 (414.0)	3202.2 (435.7)
Perinatal infant death, No. (%)	1542 (0.2)	214 (0.2)	1122 (0.2)	192 (0.2)	14 (0.3)

LMP, last menstrual period.

Neonates born to fathers aged 35–44 years had higher risks of caesarean section (RR: 1.07; 95% CI: 1.06–1.09) and PTB (RR: 1.15; 95% CI: 1.10–1.19) compared with neonates of fathers aged 25–34 years, after adjustment for confounders. Infants born to fathers aged more than 44 years had a 26% higher risk of PTB (<37 weeks) compared with neonates of fathers aged 25–34 years (adjusted RR: 1.26, 95% CI: 1.12–1.42) (Table 2), after adjustment for confounders.While the crude associations were statistically significant for caesarean delivery, SGA and PID, the magnitudes of those differences were likely not clinically meaningful (Table 2). However, the results for caesarean delivery and birth weight changed considerably between the crude and multivariable/imputed models. To test which adjustment variables were driving those changes, we separately adjusted maternal age at LMP, maternal education, first gestation, and couple’s economic pressure based on the crude model. The results showed that maternal age at LMP primarily drove those changes (Supplementary Table S2). The negative control outcome analysis revealed no statistically significant association between paternal age and secondary sex ratio in crude, adjusted or missing data imputation models (Supplementary Table S3). *E*-values for a point estimate represent the magnitude of the association that an unmeasured confounder would have to have with both the exposure (paternal age) and outcomes above and beyond measured confounding to explain away the observed association. On the basis of E-values (Supplementary Table S4), the association between paternal age (35–44 years) and PTB could be explained away by an unmeasured confounder that was associated with both the paternal age and PTB with an RR beyond 1.57. The results of sensitivity analyses were largely consistent with the primary analysis when we additionally adjusted other covariables (Supplementary Table S5). Associations between paternal age and neonatal outcomes were not modified by TTP (Supplementary Table S6).

**Table 2.** Association between paternal age and neonatal outcomes.

**Outcomes** ^a^	Paternal age at maternal LMP			
	<25	25–34	35–44	>44
	(n = 130 219)	(n = 557 671)	(n = 90 670)	(n = 5428)
**Caesarean delivery (RR, 95% CI)**				
Crude model	0.67 (0.66–0.68)	1 (reference)	1.89 (1.87–1.90)	2.07 (2.01–2.14)
Multivariable model^b^	0.91 (0.90–0.93)	1 (reference)	1.07 (1.06–1.09)	0.98 (0.94–1.01)
Missing data imputation^b^	0.91 (0.90–0.93)	1 (reference)	1.06 (1.04–1.07)	0.97 (0.93–1.01)
**Gestational age (weeks, coefficient, 95% CI)**				
Crude model	0.06 (0.05–0.07)	1 (reference)	−0.26 (−0.27 to −0.25)	−0.38 (−0.43 to −0.33)
Multivariable model^b^	−0.03 (−0.04 to −0.01)	1 (reference)	−0.09 (−0.10 to −0.07)	−0.11 (−0.17 to −0.06)
Missing data imputation^b^	−0.03 (−0.04 to −0.02)	1 (reference)	−0.08 (−0.10 to −0.07)	−0.14 (−0.19 to −0.09)
**Preterm birth (RR, 95% CI)**				
Crude model	1.06 (1.04–1.09)	1 (reference)	1.17 (1.14–1.20)	1.37 (1.24–1.51)
Multivariable model^b^	1.07 (1.04–1.11)	1 (reference)	1.15 (1.10–1.19)	1.26 (1.12–1.42)
Missing data imputation^b^	1.08 (1.05–1.11)	1 (reference)	1.14 (1.10–1.18)	1.26 (1.14–1.40)
**Birth weight (grams, coefficient, 95% CI)**				
Crude model	−12.31 (−14.63 to −10)	1 (reference)	17.88 (15.18–20.57)	19.02 (8.76–29.29)
Multivariable model^b^	−7.18 (−9.83 to −4.54)	1 (reference)	4.36 (0.76–7.96)	5.91 (−5.89 to 17.71)
Missing data imputation^b^	−7.33 (−9.83 to −4.82)	1 (reference)	4.56 (1.22–7.90)	2.37 (−8.28 to 13.02)
**Small for gestational age (RR, 95% CI)**				
Crude model	1.02 (1.00–1.04)	1 (reference)	0.87 (0.84–0.89)	0.85 (0.78–0.94)
Multivariable model^b^	1.00 (0.98–1.03)	1 (reference)	0.95 (0.92–1.00)	0.97 (0.87–1.08)
Missing data imputation^b^	1.00 (0.98–1.02)	1 (reference)	0.96 (0.93–1.00)	0.97 (0.88–1.07)
**Perinatal infant death (RR, 95% CI)**				
Crude model	0.82 (0.71–0.95)	1 (reference)	1.05 (0.90–1.23)	1.28 (0.76–2.17)
Multivariable model^b^	0.78 (0.66–0.93)	1 (reference)	1.03 (0.83–1.27)	1.00 (0.51–1.96)
Missing data imputation^b^	0.82 (0.70–0.95)	1 (reference)	0.97 (0.80–1.17)	1.15 (0.66–1.99)

LMP, last menstrual period; RR, risk ratio.

a Values are linear regression coefficients or modified Poisson regression models RR with 95% CI.

b Adjusted for maternal age, education, first gestation, and couple’s economic pressure.

**Figure 1.**
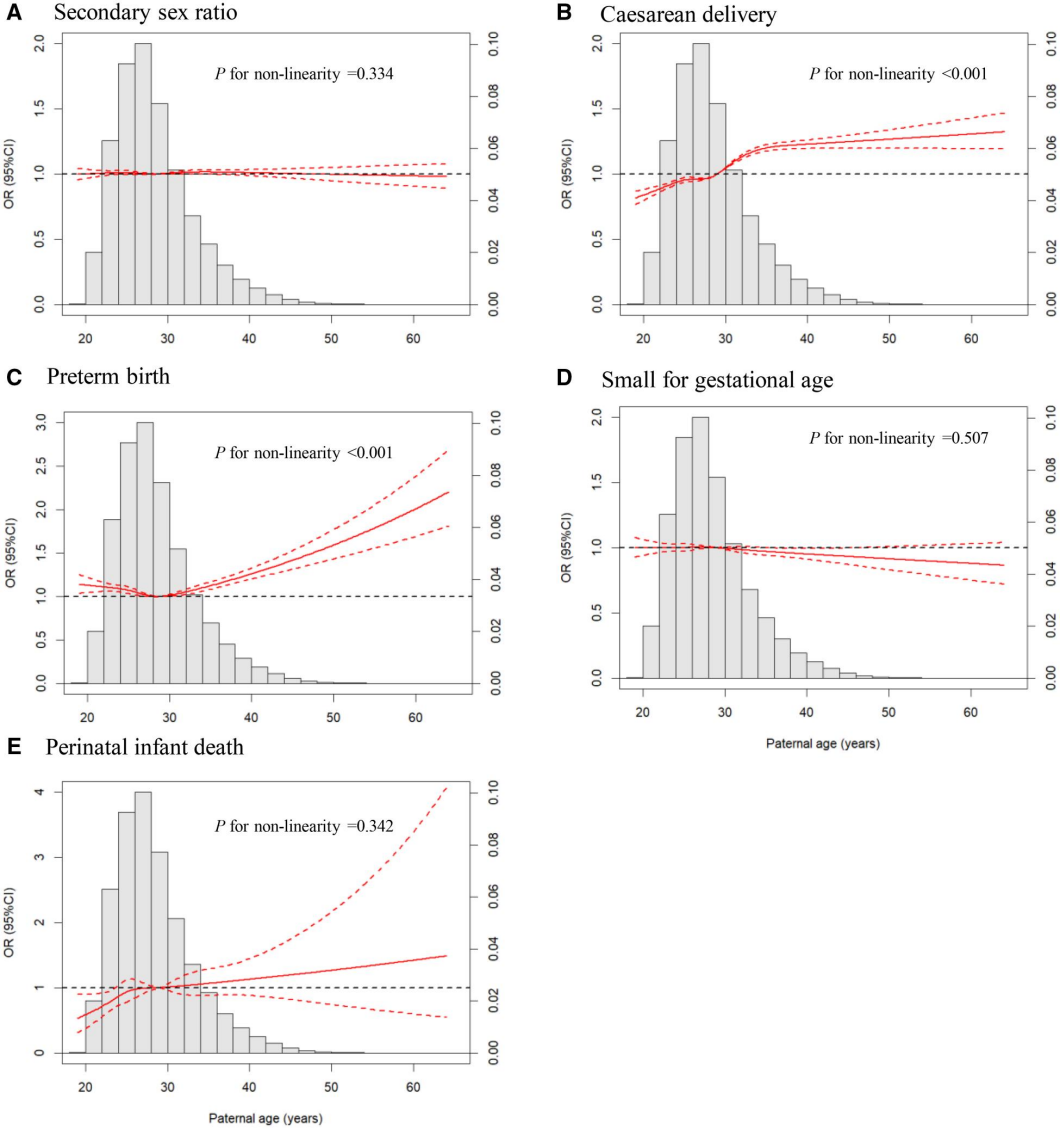
**Dose–response association between paternal age and neonatal outcomes.** (**A**) secondary sex ratio. (**B**) caesarean delivery. (**C**) preterm birth. (**D**) small for gestational age. (**E**) perinatal infant death. Adjusted odds ratios are shown by solid red lines and 95% CIs are shown by dashed red lines. Restricted cubic splines based on logistic regression models were adjusted for maternal age, education, first (vs later) gestation and couple’s economic pressure. The reference value was 29 years old, and the non-linearity of the dose–response association was tested by Wald statistics.

**Figure 2.**
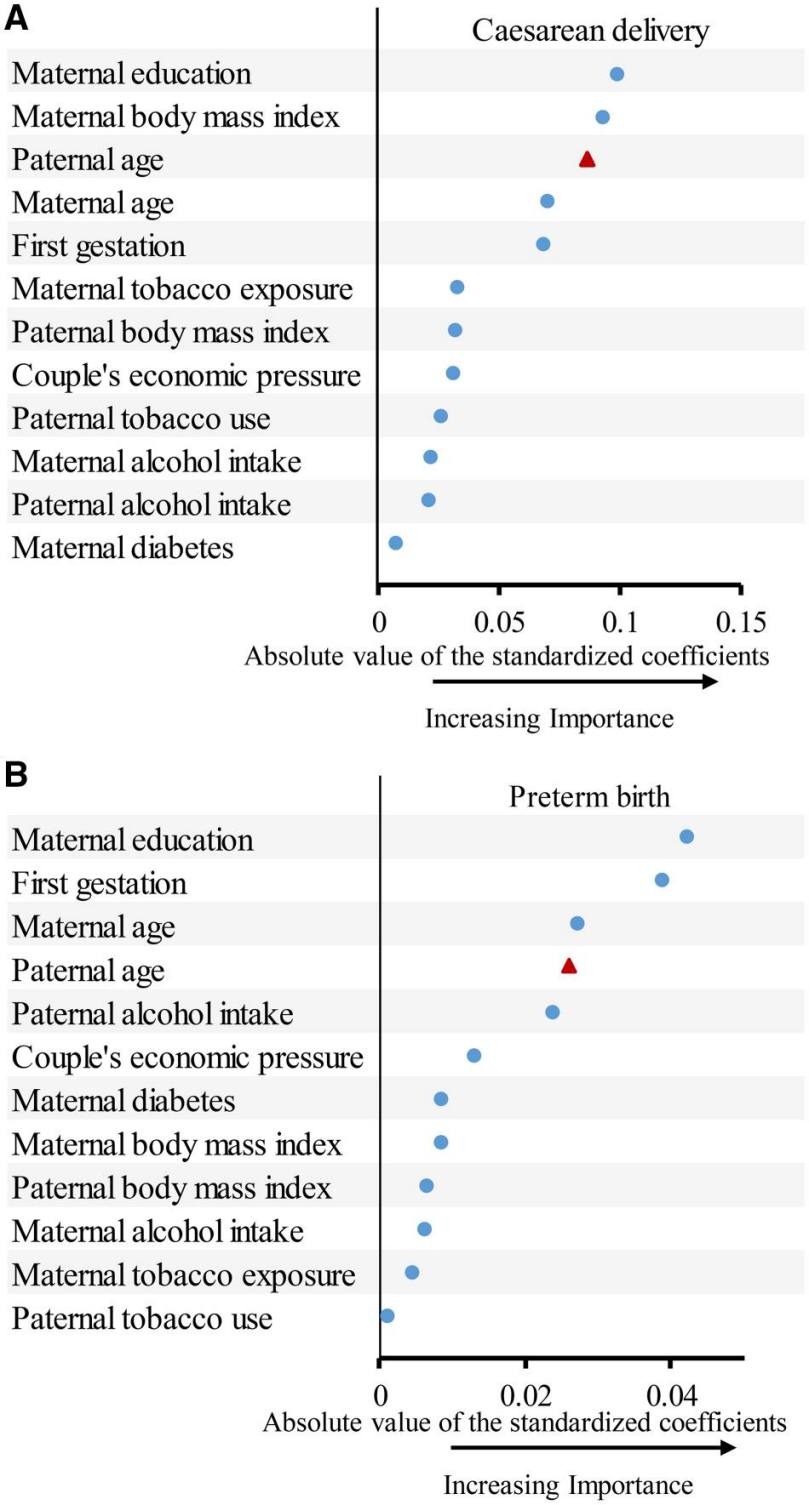
**Relative importance of risk factors for predicting caesarean delivery and preterm birth.** (**A**) caesarean delivery. (**B**) preterm birth. Relative variable importance was measured by the absolute standardized coefficients of the fully adjusted logistic model. Standardized logistic regression coefficients were computed in SAS version 9.4 (SAS Institute Inc., Cary, NC, USA) by using the *STB* option in the *MODEL* statement of *PROC LOGISTIC*.

The results of RCS analysis revealed non-linear dose–response associations of paternal age with caesarean delivery and PTB (Fig. 1B and C). After stratification by maternal age at LMP, increasing paternal age remained significantly associated with caesarean delivery and PTB, with similar trends across all stratums for maternal age except in caesarean delivery for mothers aged ≥35 years (Supplementary Fig. S3). The relative importance of paternal age was similar to that maternal age in predicting PTB, and even higher than that of maternal age in predicting caesarean delivery (Fig. 2). To validate our findings, we also examined the relative importance of different covariates using the c‐statistic (Supplementary Fig. S4) and found that the ranking of paternal age was consistent with that observed using the standardized coefficients.

Infants born to fathers and mothers who were both ≥35 years were more likely to be caesarean delivered (adjusted RR: 1.98; 95% CI: 1.95–2.00) and PTB (adjusted RR: 1.34; 95% CI: 1.29–1.40) than infants born to fathers and mothers who were both <35 years (Table 3). Joint effects for paternal and maternal age on the risk of caesarean delivery appeared to be less than additive (RERI: −0.46; 95% CI: −0.55 to −0.36). The results indicated that the joint effects of advanced paternal and maternal age were less than additive based on the estimated independent effects for each exposure.

**Table 3.** The addictive interactive effects between paternal and maternal age on caesarean delivery and preterm birth.

**Paternal age (years)**	**Maternal age (years)**	**Total no.**	**No. of caesarean delivery**	**RR (95% CI)** ^a^	**RERI (95% CI)** ^b^

<35	<35	674 155	129 997 (19.3)	1 (reference)	−0.46 (−0.55 to −0.36)
<35	≥35	5831	2442 (41.9)	1.94 (1.88–2.01)	
≥35	<35	42 038	13 286 (31.6)	1.49 (1.47–1.52)	
≥35	≥35	59 492	27 017 (45.4)	1.98 (1.95–2.00)	

**Paternal age (years)**	**Maternal age (years)**	**Total no.**	**No. of preterm birth**	**RR (95% CI)** ^a^	**RERI (95% CI)** ^b^

<35	<35	674 155	34 303 (5.1)	1 (reference)	−0.08 (−0.24 to 0.09)
<35	≥35	5831	372 (6.4)	1.30 (1.16–1.45)	
≥35	<35	42 038	2288 (5.4)	1.13 (1.07–1.18)	
≥35	≥35	59 492	3774 (6.3)	1.34 (1.29–1.40)	

RR, risk ratio; RERI, relative excess risk due to interaction.

a Models are adjusted for maternal education, first gestation, and couple’s economic pressure.

b The RERI <0 indicates less than additive joint effects.

## Discussion

This large retrospective cohort study, conducted in 783 988 mother–neonate–father trios, demonstrated that advanced paternal age is independently associated with PTB and caesarean delivery. The increased risks of PTB associated with paternal age appeared to be dose dependent, with a J-shaped association curve. Paternal age ranked similar to, or even higher than, maternal age in predicting caesarean delivery and PTB. The combined effects of advanced maternal and paternal age appeared to be less than additive joint effects. Given that many couples might not be aware of the potential impact of paternal age on neonatal health, this study underscores the importance of including, in reproductive life plans, discussions of paternal age and other risk factors that are related to age.

Consistent with previous large studies in Denmark using nationwide registers between 1980 and 1996 and in the USA using the National Vital Statistics System between 2007 and 2016 (Zhu *et al.*, 2005; Khandwala *et al.*, 2018), we found there was no change in the sex secondary ratio for older fathers. It remains likely that an altered secondary sex ratio is due to the combination of genetics and environmental exposures, which are more likely, than advanced paternal age, to explain the declining ratio of girl births (Khandwala *et al.*, 2018; Jiang and Zhang, 2021). We also found that older paternal age was not associated with an increased risk of SGA, which was consistent with most previous studies (Abel *et al.*, 2002; Sartorius and Nieschlag, 2010; Stern *et al.*, 2014; Hurley and DeFranco, 2017; Surkan *et al.*, 2019; Yin *et al.*, 2024). In contrast, one study evaluated 4621 births in the USA and found that teenage fathers were 30% (OR: 0.7; 95% CI: 0.5–1.0) less likely, and fathers older than 34 years were 70% (OR: 1.7; 95% CI: 1.3–2.2) more likely than fathers aged 20–34 years to have low birth weight babies after adjustment for child’s gender, the mother’s ethnicity, birthplace, parity, marital status, health insurance type and maternal age (Reichman and Teitler, 2006). However, this study only focused on urban disadvantaged populations. Another study that examined more than 40 million live births in the USA also found that birth weight decreased with increased paternal age (Khandwala *et al.*, 2018). However, analyses of about 0.7 million Missouri births indicated a reduced risk of SGA among infants born to fathers aged 40–45 years (Alio *et al.*, 2012). Similarly, a hospital-based study among Chinese infants also found that paternal age exerted an opposite effect on birth weight (Mao *et al.*, 2021). Moreover, one study examined more than 1 million births in Ohio from 2006 to 2012 and did not identify an association between paternal age and foetal growth restriction (birthweight <10th percentile) (Hurley and DeFranco, 2017). Therefore, more population-based studies in different populations are needed to clarify this association.

Consistent with previous studies, we found independent effects of paternal age on the risk of caesarean delivery (Tang *et al.*, 2006; Faro *et al.*, 2012; El Rafei *et al.*, 2018; Yin *et al.*, 2024). One previous study evaluated 310 574 singleton deliveries by nulliparous women and found that the overall risk for caesarean delivery was twice as high in couples where the woman was older than 35 and the man was older than 40 years, compared with couples with both parents aged 20–29 years (Tang *et al.*, 2006). It was assumed that a combination of behavioural and biological pathways may exist to link advancing paternal age and caesarean deliveries. Studies have begun to uncover the potential epigenetic link between the ageing paternal genome and neonatal outcomes (Abbasi, 2017). In mammals, imprinted genes are important in fetoplacental development and they regulate growth, morphology, and the nutrient transfer capacity of the placenta (Fowden *et al.*, 2011). Epigenetic regulation in the rapidly dividing spermatogonial cells in the testis may be disrupted as a consequence of male ageing (Bennett-Baker *et al.*, 2003). In addition, interferon-like growth factor 2 is a paternally expressed gene, susceptible to epigenetic modification, that affects growth factors for both the placenta and the embryo (Parks *et al.*, 2017). These could partially explain the increased placental weight found in pregnancies among older fathers (Strøm-Roum *et al.*, 2013), which in turn has been associated with increased risk of caesarean section (Shehata *et al.*, 2011). But a need exists to further elucidate the mechanisms between advanced paternal age and neonatal outcomes. In addition, a study suggested that fathers believe that caesarean sections are beneficial to their wives, and older fathers may be more comfortable expressing their views than younger fathers (Leavitt, 2003; Tang *et al.*, 2006). In the past few years, the Chinese government has undergone efforts to curtail the rising caesarean rate (Li *et al.*, 2017a). Therefore, national and local health commissions and clinicians have been working on reducing the caesarean birth rate, especially non-medically indicated caesarean births in China (Yan *et al.*, 2020). Our study suggests that any health policy aimed at successfully reducing caesarean section rates should include both men and women as the targeted recipients of relevant healthcare information on birth delivery modes.

Our findings were consistent with previous large population-based studies in the USA (Alio *et al.*, 2012; Hurley and DeFranco, 2017; Khandwala *et al.*, 2018), Italy (Astolfi *et al.*, 2005, 2006) and Denmark (Zhu *et al.*, 2005), which reported that advanced paternal age was associated with an increased risk of PTB. Previous hospital-based studies in China have also reported such positive associations (Mao *et al.*, 2021; Mao *et al.*, 2022; Yin *et al.*, 2024). Although the cause of PTB is multifactorial and the precise mechanism cannot be established in most cases (Goldenberg *et al.*, 2008), the association might be attributed to the following mechanisms. It was reported that the germline mutation rate is much higher in men than in women, and the difference increases with age (Crow, 2000; Goriely *et al.*, 2003). Researchers have reported that males develop ∼2 additional mutations in their germline DNA throughout life, with *de novo* mutations increasing the risk of PTB (Kong *et al.*, 2012; Li *et al.*, 2017b). The effect of paternal age on PTB may also be explained by a reduced capacity of the DNA repair system, a consequence of cumulative environmental exposures, or a selective advantage of the gametes (Zhu *et al.*, 2005). In addition, pre-eclampsia, one contributor that is known to cause PTB, has also been associated with paternal age (Harlap *et al.*, 2002; Sartorius and Nieschlag, 2010). Moreover, paternal age is not only a biological fact but also a social construct (Nybo Andersen and Urhoj, 2017). Previous studies have shown that advanced paternal age is associated with negative health behaviours (such as smoking, alcohol consumption and obesity) and medical conditions (such as subfertility), which have also been linked to adverse perinatal outcomes (Nilsen *et al.*, 2013; DoPierala *et al.*, 2016; Brown, 2018). However, whether or not the association is driven by biological or selection factors, the impact warrants attention (Brown, 2018).

### Strengths and limitations

To our knowledge, this study is the largest to investigate the association between paternal age and neonatal outcomes among the Chinese population. Moreover, our findings were strengthened by the population-based sampling framework. The NFPCP was well-established and subject to regular, standardized quality control (Zhang *et al.*, 2015; Zhou *et al.*, 2016; Xiong *et al.*, 2022), using the detailed information of 783 988 ‘mother–neonate–father’ trios for adjustment. Finally, we used modified Poisson regression models and RCS analysis so that the association between paternal age and neonatal outcomes could be comprehensively and accurately evaluated.

This study has several limitations. First, despite attempts to adjust and account for potential both maternal and paternal confounding using regression analyses and stratification, as with all observational studies, residual confounding could not be ruled out. The observed association between paternal age and adverse neonatal outcomes could be real, but it might not be causal. Second, although the NFPCP was well-implemented in Guangdong Province, with a coverage rate of over 85% of the target population (Xiong *et al.*, 2022), only couples who planned to conceive were included. Moreover, the sociodemographic characteristics, dietary factors and use of medical services in this population may not be representative of those in other regions or countries, suggesting that the findings should be validated across populations. Third, the NFPCP was designed to provide individualized preconception health advice based on preconception examination results. However, information on whether or what kind of treatment participants received afterwards was not collected. Therefore, the study may have underestimated the association between paternal age and adverse neonatal outcomes.

## Conclusions

In summary, this population-based cohort study found that paternal age was associated with an increased risk of caesarean delivery and PTB. These findings provide important evidence for preconception health care and may be clinically useful in preconception counselling regarding parental age-related pregnancy risks.

## Supplementary Material

hoaf006_Supplementary_Data

## Data Availability

The detailed personal data analysed in our study are not publicly available because of the limitations of data availability in the data management rules of Guangdong Provincial Fertility Hospital.
