# Supplementary material for: Paternal age and neonatal outcomes: a population-based cohort study
Source: Hum Reprod Open. 2025 Feb 26;2025(1):hoaf006. doi: 10.1093/hropen/hoaf006 (PMC11878789; doi:10.1093/hropen/hoaf006)
Supplement: hoaf006_Supplementary_Data [file hoaf006_supplementary_data.docx]

**Supplementary Figure S1. Flowchart of the birth cohort based on the National Free Preconception Checkup Project.**

NFPCP, National Free Preconception Checkup Project.


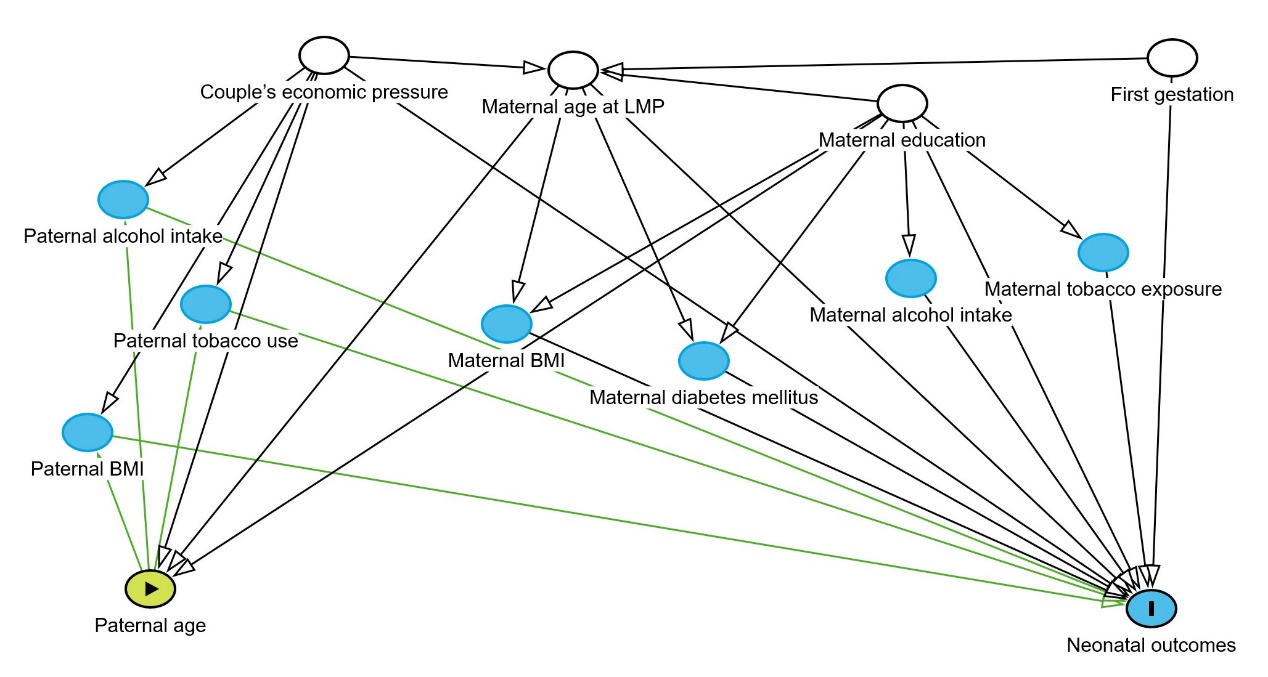


**Supplementary Figure S2. Directed acyclic graph for assessing the association between paternal age and neonatal outcomes**.

Green node, exposure of interest; navy blue node: outcome of interest; and white nodes: the adjustment set used in this study.

LMP, last menstrual period; BMI, body mass index.

**
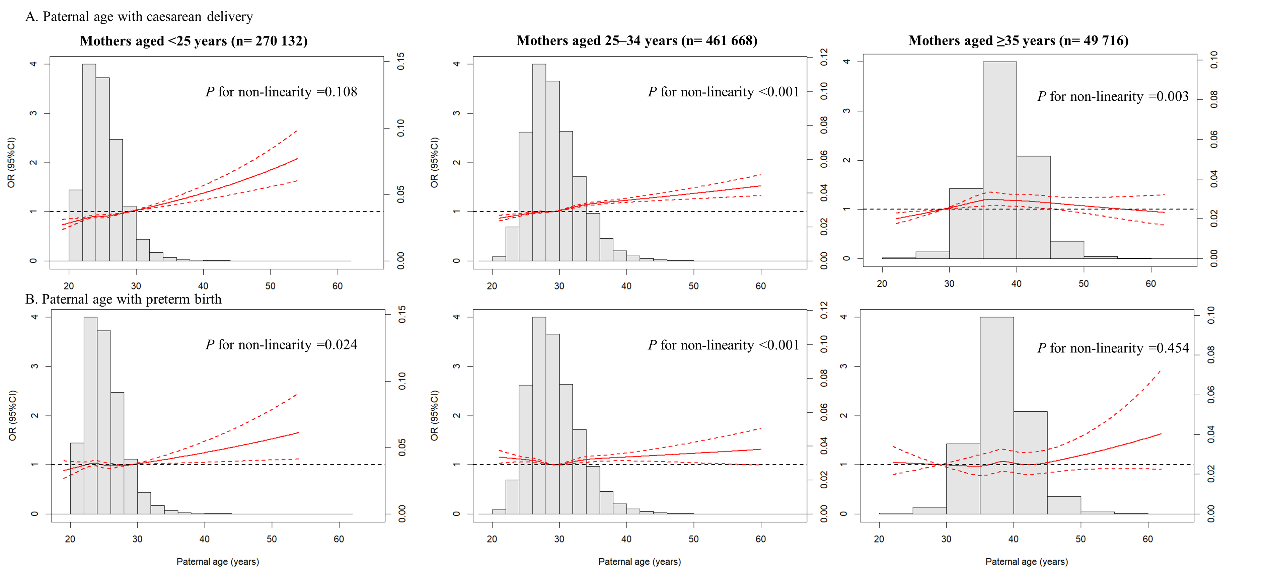
**

**Supplementary Figure S3. Dose-response association of paternal age with caesarean delivery and preterm birth, stratified by maternal age group.**

Adjusted odds ratios (OR) were shown by solid red lines, and 95% confidence intervals (CI) were shown by dashed red lines. Restricted cubic splines based on logistic regression models were adjusted for maternal age, education, first gestation, and couple’s economic pressure. The reference value was 29 years old, and the nonlinearity of the dose-response association was tested by Wald statistics.

**
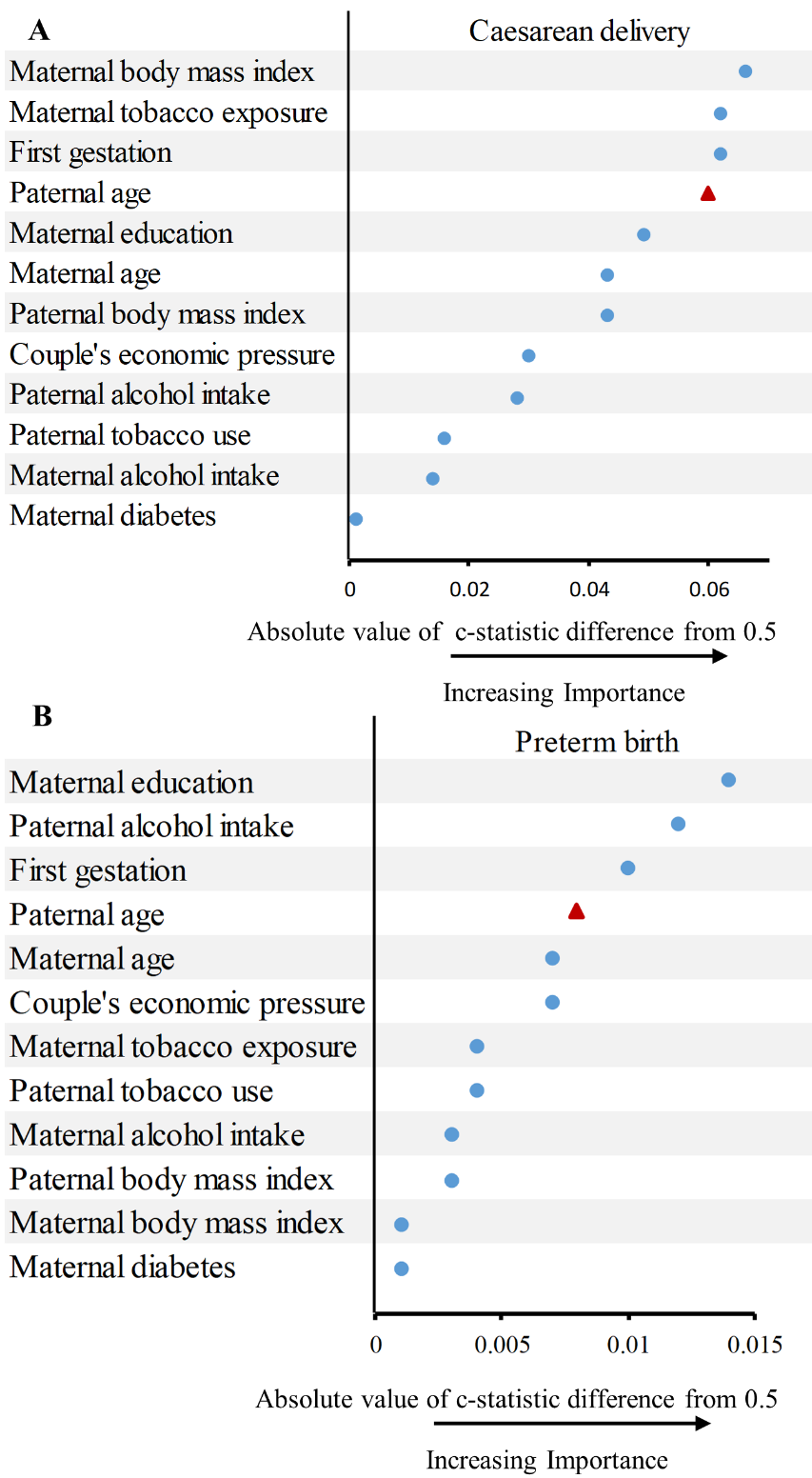
**

**Supplementary Figure S4. Relative importance of risk factors for predicting caesarean delivery and preterm birth by estimation of the c‐statistic.**

We constructed a separate logistic model for each predictor, estimated the c‐statistic for each model, then ranked the predictors in terms of the absolute value of their c‐statistic difference from 0.5.

**Supplementary Table S1. The numbers (percentages) of participants with missing covariates.**

|  | **Overall**  No. (%) |
| --- | --- |
| **Total** | 116 712 (14.9) |
| Age | 0 |
| Higher education | 92 092 (11.7) |
| First gestation | 14 243 (1.8) |
| Couple’s economic pressure | 24 022 (3.1) |

**Supplementary Table S2. Association between paternal age with caesarean delivery and birth weight after adjusting for different covariables.**

| Outcomes ^a^ | Paternal age at maternal LMP | | | |
| --- | --- | --- | --- | --- |
|  | <25 (n= 130 219) | 25–34 (n= 557 671) | 35–44 (n= 90 670) | >44 (n= 5428) |
| Caesarean delivery (RR, 95% CI) |  |  |  |  |
| Crude model | 0.67 (0.66 to 0.68) | 1 (reference) | 1.89 (1.87 to 1.90) | 2.07 (2.01 to 2.14) |
| Crude model+ maternal age at LMP | 0.90 (0.89 to 0.91) | 1 (reference) | 1.07 (1.06 to 1.08) | 0.90 (0.87 to 0.93) |
| Crude model+ maternal education | 0.70 (0.69 to 0.71) | 1 (reference) | 1.84 (1.82 to 1.86) | 2.08 (2.01 to 2.15) |
| Crude model+ first gestation | 0.69 (0.68 to 0.70) | 1 (reference) | 1.70 (1.68 to 1.72) | 1.88 (1.82 to 1.94) |
| Crude model+ couple’s economic pressure | 0.68 (0.67 to 0.69) | 1 (reference) | 1.88 (1.86 to 1.89) | 2.09 (2.02 to 2.15) |
|  |  |  |  |  |
| Birth weight (grams, coefficient, 95% CI) |  |  |  |  |
| Crude model | -12.31 (-14.63 to -10.00) | 1 (reference) | 17.88 (15.18 to 20.57) | 19.02 (8.76 to 29.29) |
| Crude model+ maternal age at LMP | -7.04 (-9.55 to -4.53) | 1 (reference) | 7.27 (3.94 to 10.59) | 4.18 (-6.49 to 14.85) |
| Crude model+ maternal education | -12.39 (-14.8 to -9.97) | 1 (reference) | 20.34 (17.47 to 23.21) | 22.71 (11.66 to 33.77) |
| Crude model+ first gestation | -10.48 (-12.83 to -8.13) | 1 (reference) | 10.92 (8.07 to 13.76) | 12.77 (2.33 to 23.21) |
| Crude model+ couple’s economic pressure | -13.21 (-15.56 to -10.86) | 1 (reference) | 17.95 (15.20 to 20.70) | 16.77 (6.21 to 27.34) |

LMP, last menstrual period; RR, risk ratio; CI, confidence interval.

^a^ Values are linear regression coefficients or modified Poisson regression models RR with 95% CI.

**Supplementary Table S3. Association between paternal age and secondary sex ratio.**

| Outcomes ^a^ | Paternal age at maternal LMP | | | |
| --- | --- | --- | --- | --- |
|  | <25 (n= 130 219) | 25–34 (n= 557 671) | 35–44 (n= 90 670) | >44 (n= 5428) |
| Secondary sex ratio (RR, 95% CI) |  |  |  |  |
| Crude model | 1.00 (0.99 to 1.01) | 1 (reference) | 1.01 (1.01 to 1.02) | 1.03 (1.00 to 1.05) |
| Multivariable model ^b^ | 1.00 (0.99 to 1.01) | 1 (reference) | 1.00 (0.99 to 1.01) | 1.02 (0.99 to 1.05) |
| Missing data imputation ^b^ | 1.00 (1.00 to 1.01) | 1 (reference) | 1.00 (1.00 to 1.01) | 1.02 (0.99 to 1.04) |

LMP, last menstrual period; RR, risk ratio; CI, confidence interval.

^a^ Values are linear regression coefficients or modified Poisson regression models RR with 95% CI.

^b^ Adjusted for maternal age, education, first gestation, and couple’s economic pressure.

**Supplementary Table S4. E-values of paternal age and neonatal outcomes.**

| Outcomes | Paternal age at maternal last menstrual period | | | |
| --- | --- | --- | --- | --- |
|  | <25 | 25–34 | 35–44 | >44 |
| Caesarean delivery | 1.43 | 1 (reference) | 1.34 | 1.16 |
| Preterm birth | 1.34 | 1 (reference) | 1.57 | 1.83 |
| Small for gestational age | 1.00 | 1 (reference) | 1.25 | 1.21 |
| Perinatal infant death | 1.88 | 1 (reference) | 1.21 | 1.00 |

E-values for the point estimate represent the magnitude of the association that an unmeasured confounder would have to have with both the exposure (paternal age) and outcomes above and beyond measured confounding to explain away the observed association.

**Supplementary Table S5. Association between paternal age and neonatal outcomes after additionally adjusted other covariables.**

| Outcomes ^a^ | Paternal age at maternal LMP | | | |
| --- | --- | --- | --- | --- |
|  | <25 (n= 130 219) | 25–34 (n= 557 671) | 35–44 (n= 90 670) | >44 (n= 5428) |
| Caesarean delivery (RR, 95% CI) | 0.92 (0.90 to 0.93) | 1 (reference) | 1.05 (1.04 to 1.07) | 0.96 (0.94 to 1.00) |
| Gestational age (weeks, coefficient, 95% CI) | -0.02 (-0.04 to -0.01) | 1 (reference) | -0.10 (-0.12 to -0.08) | -0.13 (-0.19 to -0.07) |
| Preterm birth (RR, 95% CI) | 1.07 (1.03 to 1.10) | 1 (reference) | 1.17 (1.12 to 1.22) | 1.34 (1.18 to 1.51) |
| Birth weight (grams, coefficient, 95% CI) | -5.38 (-8.08 to -2.67) | 1 (reference) | 0.52 (-3.19 to 4.22) | -0.68 (-12.91 to 11.55) |
| Small for gestational age (RR, 95% CI) | 0.99 (0.97 to 1.02) | 1 (reference) | 0.98 (0.94 to 1.01) | 1.03 (0.92 to 1.15) |
| Perinatal infant death (RR, 95% CI) | 0.79 (0.66 to 0.94) | 1 (reference) | 1.04 (0.83 to 1.29) | 0.93 (0.46 to 1.92) |

LMP, last menstrual period; RR, risk ratio; CI, confidence interval.

^a^ Values are linear regression coefficients or modified Poisson regression models RR with 95% CI. Adjusted for maternal characteristics (age, education, body mass index, diabetes, alcohol intake, tobacco exposure, and first gestation), paternal characteristics (body mass index, alcohol intake, and tobacco use), and couple’s economic pressure.

**Supplementary Table S6. Association between paternal age and neonatal outcomes across time to pregnancy (TTP).**

| Outcomes ^a^ | Paternal age at maternal LMP | | | | *P* for interaction ^b^ |
| --- | --- | --- | --- | --- | --- |
|  | <25 (n= 130 219) | 25–34 (n= 557 671) | 35–44 (n= 90 670) | >44 (n= 5428) |  |
| Caesarean delivery |  |  |  |  | 0.180 |
| TTP<12 months (n= 776 772) | 0.92 (0.90 to 0.93) | 1 (reference) | 1.07 (1.06 to 1.09) | 0.98 (0.94 to 1.01) |  |
| TTP≥12months (n= 7216) | 0.64 (0.48 to 0.86) | 1 (reference) | 0.98 (0.86 to 1.11) | 0.87 (0.68 to 1.11) |  |
| Preterm birth |  |  |  |  | 0.052 |
| TTP<12 months (n= 776 772) | 1.07 (1.04 to 1.10) | 1 (reference) | 1.15 (1.10 to 1.19) | 1.27 (1.12 to 1.43) |  |
| TTP≥12months (n= 7216) | 0.70 (0.31 to 1.61) | 1 (reference) | 1.04 (0.68 to 1.58) | 0.58 (0.17 to 2.05) |  |
| Small for gestational age |  |  |  |  | 0.845 |
| TTP<12 months (n= 776 772) | 1.00 (0.98 to 1.03) | 1 (reference) | 0.96 (0.92 to 0.99) | 0.97 (0.87 to 1.08) |  |
| TTP≥12months (n= 7216) | 1.10 (0.78 to 1.57) | 1 (reference) | 0.84 (0.62 to 1.15) | 0.79 (0.34 to 1.81) |  |
| Perinatal infant death |  |  |  |  | 0.638 |
| TTP<12 months (n= 776 772) | 0.79 (0.66 to 0.93) | 1 (reference) | 1.02 (0.83 to 1.27) | 1.02 (0.52 to 2.00) |  |
| TTP≥12months (n= 7216) | 1.61 (0.18 to 14.54) | 1 (reference) | 1.23 (0.36 to 4.19) | 1.06 (0.05 to 26.84) |  |

LMP, last menstrual period; RR, risk ratio; CI, confidence interval.

^a^ Values are modified Poisson regression models estimated RR with 95% CI. Adjusted for maternal age, education, first gestation, and couple’s economic pressure. TTP<12 months (n= 776 772) and TTP≥12months (n= 7216).

^b^ The presence of effect modification was examined by using a likelihood ratio test, comparing a model with multiplicative interaction term between paternal age and TTP to a model without the term.
